# Immune cell profiling in cancer: molecular approaches to cell-specific identification

**DOI:** 10.1038/s41698-017-0031-0

**Published:** 2017-08-15

**Authors:** Yasmin A. Lyons, Sherry Y. Wu, Willem W. Overwijk, Keith A. Baggerly, Anil K. Sood

**Affiliations:** 1 Department of Gynecologic Oncology, The University of Texas MD Anderson Cancer Center, 1515 Holcombe Blvd, Houston, Texas 77030 USA; 2 Department of Melanoma Medical Oncology, The University of Texas MD Anderson Cancer Center, 1515 Holcombe Blvd, Houston, Texas 77030 USA; 3 Department of Bioinformatics and Computational Biology, The University of Texas MD Anderson Cancer Center, 1515 Holcombe Blvd, Houston, Texas 77030 USA; 4 Center for RNA Interference and Non-Coding RNA, The University of Texas MD Anderson Cancer Center, 1515 Holcombe Blvd, Houston, Texas 77030 USA; 5 Cancer Biology, The University of Texas MD Anderson Cancer Center, 1515 Holcombe Blvd, Houston, Texas 77030 USA

## Abstract

The immune system has many important regulatory roles in cancer development and progression. Given the emergence of effective immune therapies against many cancers, reliable predictors of response are needed. One method of determining response is by evaluating immune cell populations from treated and untreated tumor samples. The amount of material obtained from tumor biopsies can be limited; therefore, gene-based or protein-based analyses may be attractive because they require minimal tissue. Cell-specific signatures are being analyzed with use of the latest technologies, including NanoString’s nCounter technology, intracellular staining flow cytometry, cytometry by time-of-flight, RNA-Seq, and barcoding antibody-based protein arrays. These signatures provide information about the contributions of specific types of immune cells to bulk tumor samples. To date, both tumor tissue and immune cells have been analyzed for molecular expression profiles that can assess genes and proteins that are specific to immune cells, yielding results of varying specificity. Here, we discuss the importance of profiling tumor tissue and immune cells to identify immune-cell-associated genes and proteins and specific gene profiles of immune cells. We also discuss the use of these signatures in cancer treatment and the challenges faced in molecular expression profiling of immune cell populations.

## Introduction

For many years, the TNM staging guidelines from the American Joint Committee on Cancer/Union Internationale Contre le Cancer have been the traditional resource for predicting prognosis in patients with various types of cancer. In recent years, however, the immune contexture of primary tumors has provided information that can be equally effective, and even superior, in predicting progression-free and overall survival. The initial studies in immune contexture were performed in colorectal cancer and then extended to ovarian, breast, prostate, kidney, head and neck, and lung cancers, and to melanoma.^1, 2^

The clinical relevance of the immune system in cancer has been shown by the growing field of immune therapy. PD-1 immune checkpoint inhibitor antibodies have been proven superior to second-line chemotherapy in achieving longer overall survival in lung cancer patients with progressive disease after initial platinum-based chemotherapy.^3^ Immune therapy is also thriving in the field of melanoma, in particular with the use of PD-1 and CTLA-4 antibodies.^4, 5^ Despite the dramatic response to immune therapies experienced by a subset of patients, discovering biomarkers to determine which patients will benefit from these drugs remains a challenge. The discovery of gene signatures has led to a preliminary model that can be used to predict response to immune therapy by evaluating gene expression in immune system cells of tumor tissue.^6^ The significance of immune profiling lies in the fact that patients in various molecular subgroups may respond optimally to different treatments.

In this article, we describe the importance of evaluating immune cell specificity with use of gene-based and protein-based analyses of tumor and immune cells and discuss the impact of such evaluations on the field of oncology. We also discuss contemporary methods of immune profiling and gene expression profiles that have been identified for major immune cell populations. Finally, we discuss many challenges in using molecular approaches to characterize anti-cancer immune responses, as well as solutions for overcoming these challenges.

## Gene expression profiling of key immune cells

Table 1 shows enriched genes, or gene expression “signatures,” identified for each individual immune cell type. These genes were identified as having the greatest differential expression (>2-fold difference) when immune cell types were compared.

**Table 1:** Enriched genes in immune cells

Immune cells	Enriched genes	References
All T cells	Human: *CD3G*,* CD3D*,* TRA*,* CD6, CD5, NPDC1, CD28, CAMK4, GFI1, GATA3, SH2D1A, TRB, TNFRSF25, NK4, TACTILE, BCL11B, CD3E, INPP4B, MAL, NPDC1, ITM2A, ITK, LCK, NFATC3, RORA, MGC19764, TCF7, ZAP70, LEF1, SPOCK2, PRKCQ, SATB1, RASGRP1, LRIG1, DPP4, CD3Z, PDE4D, FYN, WWP1, LAT, DUSP16, KIAA0748, CDR2, STAT4, FLT3LG, IL6ST*	78–79
CD8+T cells	Human: *FCGBP, C1ORF21, PHEMX, KLRG1, ZNF145, ADRB2, DUSP2, IL2RB, CCL5, GZMC, TBX21, CCL4L, GZMH, PRF1, GNLY, CST7, GPR56, KLRC1, S100B, D12S2489E, CD8B1, CD8A*	79, 81, 82
T regulatory cells	Human: *AKAP2, ANXA2, C8FW, CALM2, CDH13, ENTPD1, EPSTI1, F5, GBP2, GBP5, HS3ST3B1, HSPCA, ICA1, LAIR2, LGALS3, LOC51191, MKP-7, NINJ2, P53DINP1, PMAIP1, PTPLA, GPR2, HLA-DMA, HLA-DPA1, HLA-DPB1, HLA-DQB1, HLA-DRA, HLA-DRB4, HLA-DRB5, HPGD, PTT1, SAT, SHMT2, TMOD3, TNFRSF6, VIL2, LRRC32, CHAC1, EGR1, PYCR1, IL13, C3AR1, ZBED2, IL10, GAB3, NR4A1, IL1R2, ULBP1, IL17F, EIF4EBP1, RGS16, BCAT1, VEGFA, GIMAP1, GPR44, GPT2, PSPH, NLN, MS4A3, UCK2, TNIP3, TNFRSF9, IL7R, NR4A3, CSGALNA, CT1, SLC43A1, ZBTB32, SCFD2, HK2, C17ORF96, C1ORF186, LYZ, SFXN4, CYP1B1, SLC7A5, C17ORF58, SLC4A5, CD52, KLRB1, S100A4, POLA1, SLC7A1, ATPBD4, UBXN8, CASP8, FAIM3, IL12RB2, MPP7, ZNF831, BNIP3L, LRP8, UTRN, SOCS3, LY9, EVI2B, SFXN3, TNFRSF4, ING4, BLM, TNFSF11, SLFN5, CCR8, CD244, MYO1F, HOMER1, ATF3, AGPAT4, CTH, JAKMIP1, TFRC, RPL26L1, MICAL1, GPRASP1, TMX4, FYB, ZNF792, WDR4, HUWE1, CD83, TRIB2, LARP1B, SLC38A5, AUH, CTPS, CYTH1, APOL3, CHD6, IL1R1, LPCAT4, CITED2, DUSP4, SSBP2, LAYN, ZFAND2B, PIK3R1, GCNT4, PPP3CA, TIMP1, SIRPG, CCR3, PTP4A3, SERINC5, MAGOHB, GIMAP4, KLHL24, CCNG2, WDR70, MYBL1, CNPY4, TIMM8A, CCDC28A, MNAT1, MS4A2, GZMB, E2F5, HIBCH, C9ORF91, NHP2, CDC25B, VDR, CSF1, GPATCH4, NFKBID, TMCO7, EHBP1, FAM173B, FARS2, FLVCR2, NPM3, PNPO, P2RY8, TMEM63A, MXI1, MRTO4, SESN2, C1ORF38, STK38, SLC6A6, ALG14, LXN, TNFRSF18, ACSS1, UBA7, MTSS1, PSMA6, FAM8A1, RAB31, TNRC6B, SESN1, CEP68, LAPTM5, PLAGL2, LRRC33, EIF2B3, URB2, DDX21, ITGA4, RGS2, RORC, IFNAR2, CSDA, SORL1, LRRC37B, IL10RA, PRKACB, CBWD1, SMAD3, C1ORF96, FCER1A, HSP90AB1, EMG1*	83–87
B cells	Human: *PSEN2, FZD5, PLEKHF2, SIDT2, MTPN, TUBB6, FLJ21127, SMC6L1, KIAA1026, NUP88, GNA12, RNTRE, GSTZ1, RFX5, XYLT1, GL012, EPB41L2, LOC51760, SCRN1, PTD008, CYB561D2, SCAP2, MMP11, ERDJ5, TTC7A, PMAIP1, GARNL4, TMEPAI, SH3BP5, EDD, UCP4, EVI5L, PIK3C2B, SMAD3, LHFPL2, LOC57228, GM2A, HRK, ZNF207, LOC92497, F5, DMXL1, CLIC4, PRCP, DKFZP434C0328, STRBP, PHF16, JUP, TEAD2, PPP3CA, CNR2, ATP5B, ABCA1, BMF, ZCCHC7, KIAA0274, SLC22A3, SPI1, AMFR, ANXA4, EBF, ITPR1, HIST1H2BK, COPS3, COL14A1, SAV1, APOBEC3B, CCNG2, STAG3, LOC348938, POLD4, TBC1D1, MK2S4, APG4A, DAPP1, TNFRSF18, HERPUD1, POU2F2, ADK, CKIP-1, ACTA2, PARP14, MTSS1, DTX1, DDR1, RRAS2, RNF141, SYPL, SSPN, CORO1C, NFKBIE, CHD7, SP140, WDR34, BTNL9, ATP6V0A1, UROS, HLA-DOA, C22ORF13, IFI27, JMJD2B, WEE1, ODC1, KIAA0746, WDR11, SYNGR2, FOXP1, BLR1, MYO1E, MYBL2, GYLTL1B, SETBP1, KLF1, INPPL1, LOC54103, ZNF154, MHC2TA, CD24, ARHGEF3, BIRC3, TRIM56, HLA-DPB1, UVRAG, CD38, PEA15, FLJ10697, TLR10, ARHGAP10, HLA-DRA, SHMT2, LGALS9, FLJ10853, CEBPB, PRICKLE1, LCP21, CR1, KLHL14, TLR7, C20ORF72, HLA-DQB2, CORO1A, PRKCE* *HECW2, FLJ25604, TRIM26, FBXO41, GCNT1, LRMP, UBE2J1, IGKV3D, PCCA, GLDC, CYSLTR1, BTLA, NET5, HLA-DPA1, BLNK, CD79B, TM4SF8, TFEB, CD11ORF24,ADRBK2, CDKN2A, RIPK2, STX7, NCF4, SRGAP2, SLC2A5, CTSZ, AIM2, TCF4, TPD52, MAP3K8, MOBKL2B, HLA-DOB, IFIT3, MGC24039, BRD4, RALGPS2, SEMA4B, BSG,IGKC, HLA-DRB6, FUBP1, UNC84B, IFNGR2, HSPA5, HLA-DRB5, MARCKS, KYNU, PACAP, RHOBTB2, FLJ12363, TMED8, FGD2, FBXO10, IL4R, CD1C, MGC50844,MRPL49, CTSH, LYN, WASPIP, C3ORF6, EGR1, IGKV15, SPAP1, CHERP, IGHG3, ADAM28, HLA-DMA, HLA-DMB, PALM2-AKAP2, CD86, LAF4, LOC283663, FREB,DKFZP586A0522, UREB1, IGLL1, HLA-DQA2, KIAA1219, PLCG2, MARCH-1, BCNP1, PNOC, CD20, PSCD1, BCL11A, VPS28, SWAP70, SYK, BCL7A, NAP1L, TEM6, BTK,RAB30, FCGR2B, STAT6, BRDG1, LOC201895, ITGB1, CD74, CORO2B, VPREB3, HSPC182, MGC15619, RHOH, IGHA, CYBASC3, KIAA0125, RAM2, CD83, FCRH1, FLII,SNX10, IGHG1, EPHX1, FLJ10979, GTPAP, IGLL3, E2F5, TNFRSF17, BLK, FLJ00332, SNX2, CD200, HLA-DQB1, TRIO, CYBB, PIK3AP1, IRF4, CD22, BACE2, IGJ, PAX5,RGS13, TCL1A, MEF2C, C13ORF18, POU2AF1, HHEX, IRF8, BANK1, OSBPL10, SLC2A1, NCF1, IGL@, IGLC2, SPAP1, IGHG3, HLA-DMA, HLA-DMB, CD79A, HSPA6, IGHD,KIAA0476, SLC7A7, NKG7, CD19, SAMD9, LY86, SPIB, NAPSB, RNASE6, IGHM, LY64, CD72, IRTA2, CD1D, HLA-DQA1, SAS, CTSH, LYN, WASPIP, C3ORF6, EGR1, FREB,DKFZP586A0522*	88, 79, 89
Resting NK cells	Human: *IL18RAP, PRF1, GZMH, TKTL1*	90–92
Macrophages	^a^Murine: *Pecr, Tmem195, Ptplad2, 1810011H11Rik, Fert2, Tlr4, Pon3, Mr1, Arsg, Fcgr1, Camk1, Pld3, Tpp1, Ctsd, Pla2g15, Lamp2, Pla2g4a, MerTK, Tlr7, Cd14, Tbxas1, Fcgr3, Fgd4, Sqrdl, Csf3r, Plod1, Tom1, Myo7a, A930030A15Rik, Sepp1, Glul, Cd164, Tcn2, Dok3, Ctsl, Tspan14*	93–95
M1 macrophages	^a^Murine: *Cd38, Cfb, Slfn4, H2-Q6, Fpr1, Slfn1, Gpr18, Ccrl2, Fpr2, Cxcl10, Mpa2IOasl1, Tlr2, Ms4a4c, LOC100503664, Irak3, Hp, Itgal, Herc6, Cd300lf, Isf20, Pstpip2, Cp, Isg15, Herc6, Probe, 1452408_at, Ifi44, E030037K03, Rik, Saa3, Ifit1, Marco, F11r, Rsad2, Ddx60, Pilr1, Cpd, Gngt2, Mx1, Pyhin1, Epb4.1l3, Slfn8, Arhgap24, Nfkbiz, Gbp6, Stat1, Zpb1, D14Erd668e, Ddx58, Tuba4a, H2-T10, Ebi3, Fam176b, Xaf1, Stat2, Sepx1, Ifit2*	96
M2 macrophages	^a^Murine: *Ptgs1, Egr2, Olfm1, Flrt2, P2ry1, Vwf, Amz1, Tmem158, Tiam1, Rhoj, Mmp9, Mrc1, Bcar3, Il6st, Tanc2, Mmp12, Tcfec, Clec71, Matk, Myc, Clec10a, Atp6v0a1, Lmna, Chst7, Atp6v0d2, Emp2, Socs6, Atp6v0a1, Plk2, Ptpla*	96
Classical monocytes	Human: *S100A12, ALOX5AP, PAD14, NRG1, MCEMP1, THBS1, CRISPLD2, F13A1, MOSC1, CYP27A1, CD163, QPCT, ADAM19, ASGR2, RNASE4, ALDH1A1, EDG3, PROK2, CLEC4D, S100A9, OSM, CSPG2, GALS2, ANG, VNN2, EBI2, UTS2, RPPH1, MGST1, IL8, CCR2, SELL, DYSF, MT1F, CD14, RANSE6, CSF3R, STAB1, CYP1B1, ADHFE1, PLA2G7, MMP25, ZNF395, SLC2A3, EGR1, CMTM2, CD9*	97, 98
Intermediate monocytes	Human:* GFRA2, NKG7, PLVAP, PLAC8, MARCKSL1, E2F2, G8P4, CLEC10A, SCD, COTL1, SLC29A1, DDIT4, TGM2, LILRA3, ATF5, GPA33, C1QC, EVA1, MERTK, MGLL, DDEFL1, MARCO, NR1H3, FBP1, ACP2, GBP1, GPBAR1, SASH1, OLFM1, TIMP1, HLA-DOA, CAMK1, POUFUT1, EPB4IL3, H19, ZNF703, SNX5, CLEC10, AK-ALPHA-1, DPB1, DHRS9, MTHFD2, RGL1, PRDM1, FADS1, SLC2A8, CSK, ISOC2, CD300C, FGD2*	97, 98
Nonclassical monocytes	Human: *C1QA, HES4, CDKN1C, C1QB, RHOC, CLEC4F, ADA, TAGLN, TCF7L2, CD798, CTSL, SFTPD, ABI3, SETBP1, FGFRL1, CD798, SPRED1, SNFT, EVL, MTSS1, SH2D1B, CYFIP2, NELF, STS-1, SIGLEC10, CKS1B, PTP4A3, LYPD2, SIDT2, INSIG1, LTB, PAPSS2, ABCC3, CASP5, VOM1, HSPB1, GNGT2, HMOX1, RRAS, RNF122, CDH23, FMNL2, RGS12, SGGB3A1, FER1L3, CALML4, IFITM3, CKB, CEACAM3, IFITM1*	97, 98
Mature DC’s	Human: *ACCL5, CXCL10, CCR7, IL15, IF127, IF144L, IFIH1, IFIT1, MX1, ISG15, ISG20, IRF7, GBP4, DUSP5, NFKB1A, ATF3, TNFSF10, IL6, IL8, IL7R, CCL4, TNFA1P6, IFIT3, OASL, GBP1, HES4, CYP27B1, RIPK2, TNFRSF9, SOD2, CD38, CD44, CD80, CD83, CD86, INDO MT2A, TRAF1, GADD45B, MT1M, MTIP2, BIRC3, USP18, TUBB2A, CCL8, EBI3, IFITM1, MT1B, MT1E, MT1G, MT1H, GADD45A, CD200, LAMP3, RGS1, SAT1*	99, 100
Monocyte-derived DC’s	Human: *CD1A, CD1B, CD1C, CD23A, MRC1, CD36, HLA-DQA, HLA-DP light chain, HLA-DG protein 41, RIL, RAP1GAP, CCND2, DUSP5, PPIC, STAC, PRKACB, TRIB2, SHB*	99–101
Mature neutrophils	Human: *ALPL, IL8RB, FCGR3B, SEMA3C, HM74, SOD2, FCGR3A, IL-8, STHM, IL8RA, FCGR2A, CSF3R, NCF2, AOAH*	102, 103
Immature neutrophils	Human: *AZU1, ELA2, BPI, LCN2, MPO, CTSG, MMP8, DEFA4, DEFA3, CAMP, X-CGD*	103–105

^a^ Genes unique to mice due to lack of human specific studies

## Methods for gene expression profiling of immune cells

Initially, microarrays were used in immunology to determine the presence or absence of genes in various defined populations.^7^ Because of the large amount of data generated from a single assay, microarrays provided a means of predicting clinical outcome in cancer patients by examining gene differences between those whose disease recurs and those who remain disease-free after cancer treatment.^8^ Further analyses led to the development of gene expression signatures based on prognosis and response to treatment, including immune therapy.^8, 9^ Due to limitations, including the starting quality and amount of RNA, end point—dependent performance, minimal expression of genes due to background hybridization, and difficulty in comparing expression levels across experiments, microarrays are not widely used for gene expression profiling today.^10–14^

RNA sequencing (RNA-Seq) overcame many limitations seen with microarrays, since having prior knowledge of the sequences and being able to distinguish between highly related sequences were no longer required for performing the assay.^15^ Single-cell RNA-Seq overcame the challenges presented by the heterogeneity in gene expression in cells within the same population by enabling the investigation of single cells in order to determine their phenotype and the significance of their differences.^16, 17^ Because most non-tumor cells in the tumor microenvironment are immune cells, single-cell profiling of immune cells provides insight into the potential use of immune and other molecularly targeted therapies.^18^ Given the consistency of cytotoxic T cells in the microenvironment, single-cell analysis of T-cell phenotypes is a logical starting point for monitoring the response to immune therapy and for guiding future treatments.^19, 20^ Single-cell analysis of tumor-infiltrating T cells can illustrate varying phenotypes of T cells, including naive, regulatory, cytotoxic, exhaustion, and co-stimulatory subtypes, which can lead to predicting the type of immune therapy that the tumor is most likely to respond to.^18^

NanoString’s nCounter analysis system offers advantages over current gene expression profiling methods including digital output of data and direct mRNA measurement without enzymatic reaction.^21^ mRNA expression levels from formalin-fixed paraffin-embedded (FFPE) tissue samples, which are often degraded, can be evaluated by using the nCounter system because this system does not use an amplification step.^22^ This is advantageous for clinical trials with available FFPE samples. Genes declared absent by microarray can be detected with use of the nCounter platform, which is able to detect low-abundance mRNA with greater sensitivity.^21^ NanoString’s nCounter technologies provide immune and immune oncology-related panels of sequence-specific probes for genes of interest. Applications include identification of genes related to response to treatment, association with TIL infiltration, and tumor-specific gene alterations.^23^

The nCounter technology has been used to evaluate gene signatures in many clinical trial settings. In melanoma, a 53-gene immune signature identified by the nCounter system was able to predict non-progression, prolonged recurrence-free survival, and disease-specific survival.^24^ In breast cancer, the PAM50 gene signature was validated with use of NanoString’s nCounter platform, and was enhanced to include a risk-of-recurrence score, in addition to risk category and intrinsic subtype.^25, 26^ The nCounter system also offers the ability to analyze DNA, RNA, and protein targets at the same time using one sample. This not only allows for immune cell profiling by gene expression, but also provides protein information, providing further insight into cellular function.^27^

## Methods for proteomic profiling of immune cells

Flow cytometry is a traditional and useful method of immune cell profiling for distinguishing various populations of immune cells from a large, heterogeneous sample. Although flow cytometry is still widely used for immune profiling, newer techniques described below have advantages and can be used in conjunction with or for replacement of flow cytometry.

Cytometry by time-of-flight (CyTOF) uses heavy metal isotopes to label antibodies, and then labeled cells are analyzed by high-throughput spectrometry on a single-cell level. This approach of cell profiling provides more parameters to quantify than does traditional flow cytometry, which is limited by overlap between the emission spectra of individual fluorophores.^28, 29^ Another advantage to CyTOF for single-cell analysis of immune cell infiltration includes a required starting cell number of 1000–1,000,000, which can easily be obtained from patient biopsy specimens.^29^ CyTOF analysis has the benefit of generating valuable visualization plots to help further characterize immune populations. Analysis with SPADE (spanning-tree progression analysis of density-normalized events) yields representation of differences in signaling response seen in tight population boundaries across many immune cell types.^30^ SPADE organizes single cells into clusters of hierarchies where developmental lineages, rare cell populations, and functional markers can be easily identified.^31^

Traditional flow cytometry is fundamental in immune profiling to distinguish distinct cell populations from a heterogeneous mix. However, it is unable to track the phosphorylation of intermediate signaling molecules that play a role in immune response. Phosphoflow (also known as intracellular staining flow cytometry) overcomes the limitation of traditional flow cytometry by allowing for detection of intracellular phosphoproteins. This technique also provides single-cell analysis, thus emphasizing differences between cell populations that might appear to react similarly when probed with traditional protein-based methods such as western blotting.^32^ When combined with cell-surface staining for immune profiling, phosphoflow can provide detailed analysis of specific immune cells and their signaling pathways.^33^

Despite the benefits of traditional and phosphoflow cytometry, these modalities are unable to evaluate a single cell at multiple points in time, as the cell differentiates and evolves from a progenitor cell; this capability is an important factor in immunologic analysis. Microengraving is a technique that involves suspension of cells onto an array of microwells, inversion onto a glass slide with capture reagent, an incubation period, and then interrogation of the microarray with a fluorescence scanner.^34–36^ Microengraving is ideal for immune profiling of proteins since it allows for high-throughput identification and quantification of cell lineage and secreted products of lymphocytes, such as cytokines and antigen-specific immunoglobulins.^34, 35^ Because microengraving allows for detection of information over time, functional responses to stimulated cells, such as T cells, can be measured by serial microengraving.^36^ It is an advantageous method for studying human immune cells because it requires only 50,000–100,000 cells for analysis, and cells are retained and remain viable after analysis.^34^ Microengraving is important for characterizing secreted proteins from single cells but is unable to successfully measure cytosolic proteins.

The barcoded microchips assay has an advantage over microengraving because it allows for absolute quantification of both cytosolic and surface proteins of single cells.^35^ The microchip system contains microchambers that hold a defined volume or number of cells and houses the barcode, or antibody array for capture, lysis, and detection of various proteins.^37^ Single-cell barcoding chips require approximately 10,000 cells.^38^ In immunology, barcoding microchips have been proven useful in analyzing cytokine production from macrophages and T cells.^38^ The technique has been applied to monitoring immune therapy in the field of metastatic melanoma, where T-cell-receptor-engineered T cells, of patients participating in an adoptive cell transfer clinical trial, were profiled for 12 secreted proteins.^38, 39^ This protein profiling helps further the notion that immune response is best determined by evaluating the functional performance of the T cells rather than the number of cells present.^39^

## Clinical use of immune profiles

Cancer treatment with immune therapy, based on the molecular identification of immune cell presence and tumor antigens, has been proven beneficial in many cancers for certain subsets of patients.^40^ When immune therapy was still in its preliminary stages, the interest was in antigens that are shared among various types of tumors.^41^ It was later discovered that many of these antigens are expressed by other tissues in the host, leading to immunologic tolerance for high-avidity interactions.^40^ This limits the effectiveness of potential immune therapy due to lower-avidity immune responses.^40^ One method of overcoming this is to target neoantigens derived from point mutations in normal genes.^40^ With advances in genome sequencing, mutant antigens are being discovered that can be important for eliciting an immune response. Mutations are unique to individual tumors and can generate a more potent antitumor response by the immune system.^40^

Tumor stage, based on TNM guidelines, is a long-standing, reliable way to classify cancer. Although this system gives an estimate of the tumor burden, lymph node involvement, and evidence of metastasis, it is known that clinical outcome still varies between patients with the same stage of disease.^42^ The immune contexture of the primary tumor is an essential factor in predicting prognosis.^1^ As stated above, it has been demonstrated that knowledge of the location and density of immune cells may be more prognostic than the TNM guidelines.^1^ This highlights the misconceived notion that disease progression depends solely on tumor cells and emphasizes the importance of incorporating immune response into the classification of disease.^42^ The Immunoscore is a score from 0 to 4 generated from the density of lymphocyte populations CD3/CD45RO, CD3/CD8, or CD8/CD45RO in the tumor core and margins.^42^ The Immunoscore has been used to predict outcome in colorectal cancer by demonstrating that immune cell infiltration, as determined immunohistochemically, and its associated score directly correlate with disease-free and overall survival.^43^

The Immunoscore was recently validated in a worldwide, multi-institutional study with a primary end point of time-to-recurrence.^44^ Time-to-recurrence was significantly longer for patients with a high Immunoscore, independent of stage, sex, age, or tumor-sidedness.^44^ The immunoscore has been used successfully to predict prognosis in melanoma, breast, kidney, and lung cancers. These results, however, have yet to be validated in prospective studies in order for the immunoscore to be used as a predictive marker.^45^

## Challenges and solutions in analysis of the immunologic genome

### Gene expression signatures

#### Lack of immune-specific signatures

Gene expression signatures are used as tools to predict which patients will benefit from certain treatments and to guide decision-making in the clinic.^46^ Gene expression signatures are considered “prognostic” when they can differentiate between patients with a good or bad prognosis in the context of traditional therapy or no treatment at all.^47^ Gene signatures are considered “predictive” when they are able to predict treatment benefit between experimental and/or nontraditional treatment groups vs. control, typically in the setting of a randomized controlled clinical trial.^47^ In breast cancer, the predictive and prognostic assays Onco*type* DX, EndoPredict, PAM50, and Breast Cancer Index are now recommended as adjuncts for clinical decision-making for patients with specific subtypes of breast cancer.^46^ These signatures, however, are not specifically immune-related and share little overlap in their selected genes.^47, 48^ Because tumor cells and infiltrating immune cells both have prognostic value, evaluating tumors and the surrounding stroma with use of the methods described above, in order to generate immune-related signatures, can provide prognostic and perhaps predictive information associated with patient outcome. Although there are no immune-related gene signatures used in clinical practice currently, several studies have shown the validity and reproducibility of using immune-related signatures to predict outcome and response to therapy in patient cancer samples.

#### Solution: in silico, pan-cancer studies

One reason immune-related gene signatures have not been widely used in clinical settings is the lack of consistency of genes, both within the same tumor type and among different tumors.^49^ Efforts to address this problem are under way, with the development of conserved immune gene signatures representing multiple tumor types.^49^ Another problem with immune-related gene signatures is the difficulty in deciphering whether the genes represent specific immune cell populations, antigen-presenting machinery, cytokines, or other immune-related molecules. In silico studies have validated pan-cancer immune-related gene signatures that represent many immune cell types with known prognostic significance including T cells, B cells, NK cells, macrophages, as well as various cytokine populations.^49, 50^ Immune-related gene signatures have proven to be prognostic of clinical outcome across multiple cancer types and are associated with response to immune therapy in specific cancers.^49, 50^ Recent findings on immune-related gene signatures have shown that gene signatures are able to distinguish between broad lineages of cell type, such as lymphoid and myeloid but are less adept at distinguishing between more differentiated cells such as CD8^+^ and CD4^+^ T cells.^49^

### Ranked gene lists

#### Oversimplification of interpretation

Currently, given the multitude of methods with which to analyze genomic data, the problem no longer lies in obtaining gene expression profiles but rather in interpreting the data. Analysis of gene expression data typically involves a comparison of two groups and the creation of a ranked gene list based on expression. One method of interpretation involves looking at each end of the ranked list to evaluate genes showing the largest difference in expression.^7, 51^ Problems with this method include small relevant biological differences between genes leading to no statistical significance, a long list of genes that are statistically significant but lack biologic relevance, missed effects on pathways by analyzing single genes, and variation in gene lists for the same biologic system based on two different groups’ data.^51^ This again underscores the challenge faced when trying to create a clinically useful immune-related gene signature from a high-throughput panel of genes.

#### Quantity of data

The NCBI Gene Expression Omnibus database contains more than 30,000 series and 1 million samples of array-based expression data, many of which involve the immune system.^52^ Because of the large amount of expression data relating to the immune system and the inability to compare information between the various datasets, the Immunological Genome Project (IGP) was created to provide a comprehensive compendium of the expression of protein-coding genes for all immune cell populations in the mouse immune system.^53^ This project was developed to provide information on primary immune cells isolated ex vivo from the mouse immune system and then integrate that information into networks, while taking into account variation via genetic polymorphisms, knock-out of genes, knockdown via RNA interference, and drug treatment.^53^ Researchers can use these data to determine gene expression profiles that serve as biomarkers for predicting response to treatment and for measuring residual disease after immune therapy.

#### Solution 1: added-value/specialty databases

Primary databases contain gene expression and other genomic data such as genotype, DNA methylation, and protein expression data. These databases are complex, and bioinformatics expertise is often required to process the data.^54^ Added-value databases take information from primary databases and answer a question related to comparing two groups. The “added-value” involves additional data processing, mapping to standardized vocabularies, and additional annotation and analysis.^54^ An example of an added-value database includes the Gene Expression Atlas, which provides gene expression data across various cell types, organisms, developmental stages, and disease states.^55^ This atlas also includes information on gene expression in treatment and other experimental conditions.^55^

Specialty databases, such as immunology databases, have been designed to yield more information about a particular topic and its related biology. In immunology, because of the highly conserved nature of immunologic proteins, even a slight variation in sequence can produce a significant biologic effect.^56^ This calls for a more comprehensive analysis of the genetic variations in this population, found in a specialty database for immunology.^56^

#### Solution 2: co-expression databases

Co-expression data between two genes can be useful in studying gene function, protein interaction, and signaling pathways and can provide information on predicting survival and determining biomarkers.^57^ ImmuCo is a cell-specific, co-expression database that provides information between any two genes in immune cells.^57^ Traditional methods of identifying gene co-expression and correlation include Pearson and Spearman correlations; however, under certain conditions, these methods do not represent the same relationship. In ImmuCo, gene co-expression is represented by signal values and detection calls, with signal values indicating gene expression level and detection calls representing gene coexistence.^57^ ImmuCo provides data on cell-specific gene co-expression by plotting the genes on either axis of a scatter plot in a given cell type.^57^

Immuno-Navigator is both a gene and co-expression batch-corrected database for 24 mouse immune system cells.^58^ Batch effects are technical sources of variation in data that behave differently across various experimental conditions and are not related to the biology of a study; they are particularly pronounced in co-expression databases.^58, 59^ Removal of batch effects improves consistency between cell type-specific expression correlations.^58^

### Overcoming the challenges of using small tumor samples

#### Using paraffin-embedded tissue

A hallmark of personalized medicine with immune therapy is the use of outcome-related clinical data to drive hypotheses. Therefore, archived clinical samples such as FFPE are of great value when fresh frozen tissue does not exist. Methods to improve the use of these long-term samples are still needed. Using fresh frozen tumor samples to isolate RNA and perform microarray analyses has been a long-standing basis for genome-wide profiling.^60^ Efforts to increase the use of FFPE samples for genomic studies are important, since fresh frozen tissue is not as accessible or as easily stored.

Advancements in technology have enabled more widespread use of FFPE samples, yielding alternative tissue available for genomic studies aside from fresh frozen tumor. FFPE samples have been used to evaluate the feasibility and accuracy involved in identifying a breast cancer prognostic gene signature previously identified with use of fresh frozen tumor.^48^ The breast cancer gene signatures listed above are now approved for use with FFPE tissue.^48^ FFPE tissue closely correlated with the fresh frozen samples in its ability to predict good vs. poor prognosis, with *r* values of 0.88 and 0.81, respectively.^60^ To establish whether RNA isolated from FFPE samples was of sufficient quality to be used to discover gene signatures, the gene expression data were validated against the fresh frozen samples. Errors seen in gene signature data were likely secondary to poor-quality FFPE samples, and when these samples were removed, the error rate was <10%.^60^ This suggests that data obtained from FFPE tissue can predict the same gene expression signatures as fresh frozen tissue, with minimal error. In addition to RNA isolation from FFPE tissue, efforts are now being made to isolate protein from the same tissue, which has proven to be difficult in the past due to cross-linking that occurs after formalin fixation.

#### Using high-throughput analyses

Tissue microarrays (TMAs) allow the assessment of biomarkers for hundreds of patients at a time while minimizing labor and time. However, because of the reduced amount of tissue sampled, TMAs must reflect the overall architecture of the tumor, including the immune contexture. Using TMAs, a map of the tumor landscape can be created to represent the density and distribution of immune cells in various regions of the tissue, including the center of the tumor vs. the margin.^61^ Although this type of map can be created on an individual-patient basis, the massive throughput capacity of TMAs allows for generalization of the immune architecture in a specific tumor type. This can facilitate Immunoscoring in these patients to strategize individualized treatment. When evaluating the heterogeneity of TMA samples compared with traditional large-section slides, TMAs had similar concordance with large sections, ranging from 70 to 95%, depending on the protein of interest.^62^

### Heterogeneity within cell populations

Variations in gene and protein expression within an individual population of cells can have consequences when creating cell-specific markers. The molecular differences within immune cells can be seen at the DNA, RNA, and protein levels. Although cell-to-cell variability can have a detrimental effect on phenotype, the immune system uses this variability to its advantage. Variability in DNA is used by the immune system to respond to a diverse set of antigens.^63^ For example, the V(D)J site-specific DNA recombination process generates a multitude of T-cell and B-cell receptors that are essential for host immune defense.^63^ At the RNA level, variability between cells can be classified as “intrinsic,” due to stochastic processes, given the finite amount of nucleic acid and regulators present in cells, or “extrinsic,” due to factors upstream of mRNA synthesis that can have an impact on cellular function.^63^ At the protein level, immune cell populations exhibit cell-to-cell variation in key factors of cellular function such as signaling molecules and transcriptional regulators.^63^ This variation contributes to the plasticity of immune response, for example, by affecting antigen response during T-cell activation.^64^ Overall, heterogeneity within immune cell populations on DNA, RNA, and protein levels is an advantage of the immune system, and the computational and experimental techniques listed below are crucial in understanding these differences.

#### Solution 1: ontologic-structured databases

Analyzing gene expression within an ontological framework allows for new ways to understand cell heterogeneity and appreciate cell identity.^65^ The ontologically based molecular signature (OBAMS) method uses IGP data to generate new biomarkers for immune cells by incorporating the information into an ontology.^65^ Constructing ontologies allows a hierarchical approach to identifying cell markers. Data from IGP generates genes specific to B cells vs. other lymphocytes, and gene expression varies greatly depending on the type of B cell.^65^ Using OBAMS, cellular biomarkers are differentiated at every level of cell identity, creating a pyramid from very broad to very specific classifications. Aside from cell identity, biologic function can be ascertained via term enrichment from several ontologies, generating new ideas of biologic function in cell types that are independent of experimental evidence from the literature.^65^

#### Solution 2: single-cell profiling

Several single-cell profiling techniques for both RNA and protein studies were described above. These methods help improve the detection of rare cell populations, explore the maturation process of certain immune cells, and emphasize the individuality of cells from the same lineage. For example, T cells derived from the same progenitor, but bearing different T-cell receptor sequences, express different secretory factors and differentiate into different types of mature T cells.^66^ Single-cell profiling using RNA or protein-based methods can further characterize these differences and provide functional information regarding these populations. Characterization of epigenetic changes, including methylation and chromatin modification, can also provide insight into variations in phenotype between cells.^63^

## The overall approach

The overarching goal of immune cell molecular studies from clinical tumor samples is to understand the interactions between the host immune system and the tumor. This allows for the generation of hypotheses that focus on prognosis, pharmacodynamics, measurement of residual disease, and prediction of patients who will respond best to immune therapy.

When comparing gene expression differences before and after immunotherapy, the most important issue begins with the type of tissue to collect for RNA extraction, since this is the key step for molecular profiling of the tumor. For proteomic studies, having a high concentration of protein before processing is most important for minimizing sample loss.^67^ Depending on the organ system being studied, tissue samples can vary widely. Blood cultures are less heterogeneous but may not be the best choice for studying cancers outside of the hematologic malignancies. However, in certain situations, blood can serve as a surrogate for various disease sites when other tissue is not available. Some disadvantages of other tissues include the fatty nature and lack of cell content in breast tissue, the fibrous nature of muscle, and the high cell density of the liver.^68^ Tissue from the ovary is heterogeneous; therefore, certain sections might not represent the larger tumor. In addition, full-thickness tissue, such as skin, requires mechanical destruction and can lead to disruption of the sample.^68^

Immediate freezing of surgically removed tumor tissue is superior to FFPE for preserving RNA; however, the availability of fresh frozen tissue is typically limited whereas paraffin embedding allows for years of storage. Tissue quantity also plays a role in quality of RNA extracted, with smaller amounts of tissue having higher-quality RNA; for example, 1–5 mg of tissue showed higher-quality RNA in gastric, liver, and muscle tissue than did larger quantities (5–15 mg) of the same tissue samples.^68^ In addition to improving RNA quality, samples that were less than 1 cm improved real-time polymerase chain reaction amplification 5-fold.^69^ Historically, protein is extracted from fresh frozen tissue or blood but not from FFPE tissue due to cross-linking that occurs upon formalin fixation.^70^ Recently, methods for extracting protein from both FFPE tissue samples and samples frozen in optimal cutting temperature (OCT) compound have been described, and protocols are available.^70, 71^

Although blood and cells are superior to tissue for processing and collecting high-quantity and high-quality RNA, the practicality of using FFPE tissue and fresh surgical specimens emphasizes the need for sufficient processing of a variety of tissue. RNA isolation and purification kits and methods of processing have evolved to yield greater quantities of RNA. These modifications include the addition of a ribonuclease inhibitor for ribonuclease-rich tissue such as the pancreas, continuous extensive mincing of skin biopsies under liquid nitrogen, and fixation with 30% sucrose for liver tissue with microwave fixation/processing.^72–74^ Due to advancements in RNA isolation and purification, a variety of cancer tissues that were previously difficult to process can now be processed for RNA extraction with use of specific isolation kits. The specimen should be approximately 1 cm or smaller and weigh less than 5 mg in order to yield the highest quantity and quality RNA. Because of newer technologies such as NanoString, which provide DNA, RNA, and protein information from the same sample, techniques that enable simultaneous extraction of all three molecules are necessary. Protocols using human cells in culture have shown comparable extraction of all three molecules compared with control methods. One protocol involves pelleting of the cells, the addition of saturated phenol for separation of all three molecules, and the addition of chloroform for phase separation. At the time of phase separation, further techniques are used to isolate RNA from the upper phase, DNA from the middle and lower phases, and protein from the lower phase.^75^

## Conclusions

It is well known that tumor cells express antigens that can be recognized by the immune system to provoke an antitumor response. Despite this immune response, many patients still have progressive disease that fails to respond to immune therapy.^40^ This underscores the importance of using the cancer immune phenotype to tailor therapy. Molecular profiling of the immunologic genome provides methods for classifying and representing the immune cells at the gene and protein level, while also defining groups and networks of molecules with similar functions from similar or different lineages.^53^ In studies of expression profiling of immune cells as an immune signature, significant differences in overall survival have been shown in a variety of cancers.^76^ Future directions in the field of cancer immunotherapy, especially in terms of precision and personalized medicine, include “next-generation functional tests,” which use baseline genomics and the interactions of all cellular components (DNA, RNA, protein) to determine live-cell sensitivity to drug treatment that can be administered rapidly.^77^
